# Smoking and Pancreatic Cancer: Smoking Patterns, Tobacco Type, and Dose-Response Relationship

**DOI:** 10.7759/cureus.26009

**Published:** 2022-06-16

**Authors:** Muhammad Subhan, Nisha Saji Parel, Parimi Vamsi Krishna, Anuradha Gupta, Kamsika Uthayaseelan, Kivonika Uthayaseelan, Monika Kadari

**Affiliations:** 1 Internal Medicine, Jinnah Hospital, Allama Iqbal Medical College, Lahore, PAK; 2 Family Medicine, Tbilisi State Medical University, Tbilisi, GEO; 3 Internal Medicine, Jagadguru Jayadeva Murugarajendra (JJM) Medical College, Davanagare, IND; 4 Research, Government Medical College, Nagpur, IND; 5 Internal Medicine, All Saints University College of Medicine, Kingstown, VCT; 6 Internal Medicine, All Saints University School of Medicine, Roseau, DMA; 7 Internal Medicine, Bhaskar Medical College, Hyderabad, IND

**Keywords:** smoking and cancer, smoking patterns and pancreatic cancer, tobacco and pancreas, cigarette and other tobacco products act, smoking and pancreatic cancer, pancreatic cancer, quitting smoking, smoking cessation, cigarette smoking, smoking tobacco

## Abstract

Pancreatic cancer (PC) is the primary cause of cancer death in the United States and Europe. Despite remarkable advances in the molecular understanding of PC and advances in new therapeutic approaches, PC remains a disease with a poor prognosis. Although evidence indicates that long-term smoking is a major cause of PC, the molecular pathways behind smoking-induced PC pathogenesis are not fully understood. Smoking cessation can significantly reduce the occurrence of PC.

This review explores the processes underpinning the influence of smoking-related chemicals on fibrosis and inflammation and provides insight into the etiology of PC. In the future, a thorough exploration of the effects of smoking chemicals on the activity of pancreatic stem cells and then on the essential mediators of the association with cancer cells would likely yield new diagnostic targets.

## Introduction and background

The pancreas is a retroperitoneal organ in the abdomen that performs exocrine and endocrine functions [1]. It is an oblong organ located at the level of the transpyloric plane at the level of lumbar vertebra 1 [1]. Pancreatic tumors can be non-endocrine or endocrine. The benign tumors include adenoma, cystadenoma, lipoma, and fibroma while malignant tumors include ductal adenocarcinoma and cystadenocarcinoma [1]. Giovanni Battista Morgagni's 1761 work 'de Sedibus Et Causis Morborum Per Anatomen Indagatis Libri Quinque' was the first recognized description of pancreatic cancer (PC) [2]. PC is the world's twelfth most widespread malignancy and is the fourth most common cause of mortality [3]. According to Globocan predictions, there will be 640,500 diagnoses and 606,300 deaths due to PC worldwide by 2030 [3]. PC incidence and mortality rates are rising due to the aging population worldwide. Furthermore, males are statistically more likely to get the disease than females [4]. In the previous few years, there has been an increase in PC incidence and fatality rate, irrespective of sex [5]. The Surveillance, Epidemiology, and End Results (SEER) statistics by the National Cancer Institute between 2000 and 2014 show an age-specific trend toward an increase in PC incidence in two specific age groups (20-29 and over 80 years old) in the United States [6]. Caucasian patients had a higher incidence and fatality rate than black patients [6]. Advancing age is another risk factor for PC, with one in 61 individuals developing the disease by 85 years old [6]. The risk factors for PC are summarized in Table 1 [7-8].

**Table 1:** Risk factors of PC PC: pancreatic cancer

Modifiable risk factors	Non-modifiable risk factors
Tobacco smoking	Increasing age
Alcohol intake (>4 standard drinks/day)	Familial cancer syndromes BRCA1 gene and BRCA 2 carrier status
Obesity	Afro-American race
Non-vegetarian diet	Non-O blood group type
Toxins (pesticides, benzene, certain dyes, and petrochemicals)	Hereditary and chronic pancreatitis
	Diabetes and cystic fibrosis

PC arises from microscopic (pancreatic intraepithelial neoplasia) or macroscopic (pancreatic cystic precursor lesions) lesions that may be intraductal papillary mucinous neoplasm and mucinous cystic neoplasm (MCN) [9]. Weight loss accompanied by back or upper abdominal pain, diarrhea (especially steatorrhea), loss of appetite, constipation, dyspepsia, nausea, vomiting, and new-onset diabetes are common symptoms in patients over 60 years old [10]. The diagnosis of PC is primarily confirmed with serum cancer antigen 19-9, a serum marker for PC authorized by the US Food and Drug Administration (FDA) [11]. Computed tomography, magnetic resonance cholangiopancreatography (MRCP), and endoscopic ultrasonography are the current diagnostic techniques for suspected PC and screening of people at high-risk [12]. PC treatment varies depending on patient factors like age, body mass index, lifestyle, personal preferences, clinical stage, and other morbid complications [13]. The quintessential steps to manage PC involve nutrition and pain management, biliary drainage, surgery, radiotherapy, chemotherapy, and ablative therapy [13]. Pancreaticoduodenectomy, distal pancreatectomy, and total pancreatectomy are the three major pancreatic resections, with the type of resection chosen by the tumor's location. These methods have become standardized regarding the extent of resection and lymph nodes extracted [14]. PC is a severe disease with a fatality rate that mirrors its epidemiology, and most patients with PC are asymptomatic until the disease has progressed fatally. Smoking has been analyzed as a risk factor for PC, as the generation of toxic metabolic by-products derived from tobacco and nicotine damages the pancreatic ductal cells. This review aims to explore the associated risk of tobacco smoking with PC progression and how its cessation can reduce the risk and occurrence of PC.

## Review

PC is the fourth major cause of mortality in the United States and the sixth in Europe [15]. PC affects 44,000 Americans each year and at least 250,000 people globally [16]. The causes of PC are relatively unknown, despite several risk factors having been identified [5]. Smoking is the most potent recognized risk factor for PC [16-18]. According to Lynch et al., current cigarette smokers have an 80% increased chance of PC than those who have never smoked [18]. Cases are expected to rise internationally because of the growing aging population and widespread adoption of cancer-causing habits such as cigarette smoking (CS), cigar-smoking, and smokeless tobacco [17]. Due to the increased mortality rate and poor screening of PC, there is a need to summarize the current statistics between tobacco and PC. This article provides updated statistics on PC based on etiology and identifies the main risk factors primarily tobacco in the progression of PC.

Molecular mechanisms of smoking-induced PC

Even though most studies have focused on the dangers of CS, tobacco in any form is dangerous [19-23]. Smokeless tobacco, like smoking tobacco, is also known to cause PC in humans [23]. At least 250 out of 7,000 chemicals found in tobacco smoke are harmful, and 60 out of them are carcinogenic, including arsenic (As), Nickel (Ni), and benzene (C6H6) [24-26]. Some different nitrosamines such as “4-methyl-nitrosamine-1,3-pyridyl 1,butanone (NNK), N′-nitrosonornicotine, and 4-methyl-nitrosamine 1,3 pyridyl 1,butanol” [26]. NNK (a nicotine metabolite) is among the most common carcinogens in cigarette smoke [26].

**Figure 1:**
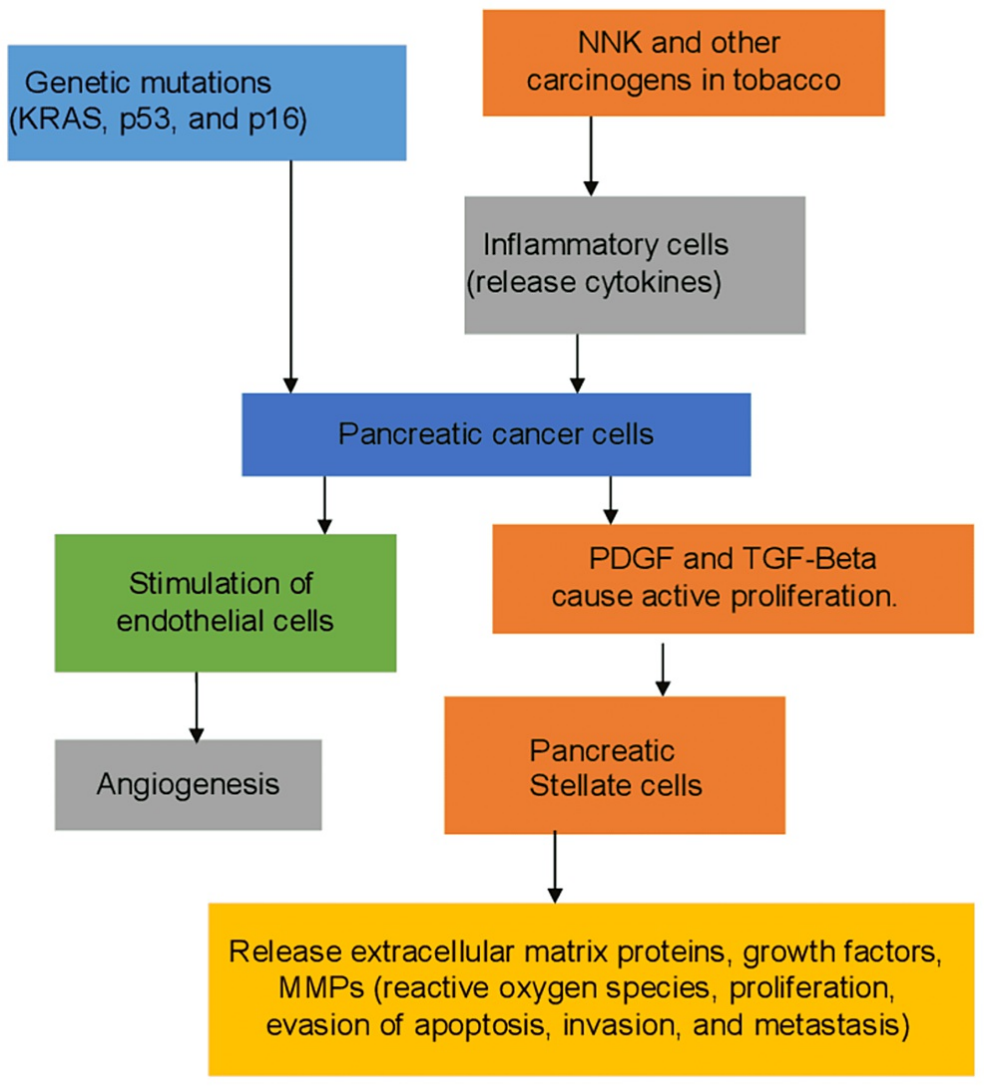
Molecular pathogenesis of PC PC: pancreatic carcinoma; NNK: nicotine-derived nitrosamine ketone; PDGF: platelet-derived growth factor; TGF-Beta: tissue growth factor-beta; MMPs: matrix metalloproteinases Image credits: Muhammad Subhan

Although overwhelming evidence shows that heavy cigarette smoking is a major risk factor for PC, the exact processes behind PC pathogenicity caused by smoking are unknown [27]. Due to CS, pancreatic acinar cells show hyperplasia and dysplasia [19]. Acinar cell damage in laboratory mice exposed to tobacco smoke was similar to that found in humans [20]. Therefore, it was indicated that this led to acinar cell cancer, as CS caused nuclear atypia ( abnormal appearance of cell nuclei) in glandular cells [21]. In a study that looked at the histochemical effects of CS on rats, the pancreatic islet cells showed a considerable decrease in insulin hormone release. In contrast, the pancreas showed increased glucagon expression [22], indicating that smoking may have a diabetogenic effect [22]. This is indicative of the fact that chronic tobacco users are generally glucose intolerant when compared to non-smokers [22]. Accumulating evidence suggests that carcinogenic compounds in CS promote PC progression by stimulating inflammatory and fibrotic changes, which work in tandem with other factors that cause gene mutation to stimulate the proliferation of glands [23]. Now confirmed as the central participant in pancreatic fibrogenesis, pancreatic stellate cells (PSCs) are the leading cause of cancer desmoplasia [28]. PSCs are dormant or quiescent cells in the normal pancreas [29]. PSCs are activated to a myofibroblastic state with pancreatic damage, such as tobacco, leading to the production of excessive amounts of ECM proteins [29]. Different nitrosamine compounds and nicotine of CS in smokers' pancreatic juice interlink their strong association with PC [30]. Previous studies show that nicotine induces an aberrant manifestation of the MUC4 mucin in PC with its progression and metastasis [27]. Momi et al. surveyed the pathogenesis of PC due to smoking, which has shown that nicotine (an ingredient in tobacco smokes) causes the upregulation of *muc4* [27]. “This nicotine-mediated *muc4 *overexpression was via the α7 subunit of nicotinic acetylcholine receptor (nAchR) stimulation and subsequent activation of the JAK2/STAT3 downstream signaling cascade cooperation with the *mek/erk1/2* pathway” [27]. Nicotine-mediated *muc4* activation promoted PC cell migration by stimulating downstream signaling such as “*her2, c-src*, and *fak*”. It was diminished by short hairpin ribonucleic acid (RNA)-mediated *muc4* abrogation, suggesting that nicotine's molecular effects on PC cells depend upon *muc4* [27]. NNK was found in PC cell growth by activating Cox2 via the adrenergic receptor [31]. Furthermore, NNK transactivates epidermal growth factor receptors, increases the intracellular level of cyclic adenosine monophosphate, and activates phosphorylation of the *erk* gene in glandular cells of the pancreas by interacting with adrenergic receptors 1 and 2 [32]. Other studies have shown that *kras* mutation, a significant mutation in patients with PC, led to the upregulation of downstream genes such as the *braf, mek, and erk* mediated rapid growth of glandular cells [23]. Some mutations, such as the ttn gene, were more prevalent in the pancreas of smokers than in non-smokers [33]. Thus, nicotine induces different changes at the molecular level that causes hyperplasia of pancreatic glandular tissues. The different molecular mechanisms that lead to PC by various genetic mutations and carcinogens are summarized in Figure 1.

Tobacco as a risk factor: types and dose-response relationship

Smoking has been shown to harm prognosis in individuals with cancers pathologically linked to CS use such as the lungs, gastrointestinal tract, and head and neck cancer [34]. However, it remains unclear whether CS leads to a worse clinical course and impacts prognosis in people with PC.

A study from the “Pancreatic Cancer Cohort Consortium (PanScan)” reported that current smoking had an odds ratio of 1.77 with 95% CI 1.38-2.26 for PC [18]. Hence, this study reported smoking as a significant risk factor for PC and that people who smoke are more likely to have PC than non-smokers. The above research can be compared with a study performed by Iodice et al. in 2008 that included 82 published studies between 1950 and 2007 that showed that the risk of PC for present and past smokers was 1.74 with a 95% CI of 1.61-1.87 and 1.2 with a 95% CI of 1.11-1.29, respectively [35]. Hence, PC presents in both former and current smokers. Compared to non-smokers, smoking cigarettes increased the risk of PC by 75% [35].

PC is most commonly associated with CS. There has been limited evidence that other tobacco-containing items, including smokeless tobacco, cause PC [36]. Bertuccio et al. performed another pooled analysis in 2011. This study associated CS, pipe smoking, use of smokeless tobacco, and the number of cigars consumed per day with the association of PC. The OR for cigar-only smokers was 1.6 while the 95% CI was 1.2-2.3 compared to patients who never used tobacco. Thus, cigar smokers were 1.6 times more likely to have PC than patients who had never used tobacco. For cigarette smokers, the OR was 1.5 while the 95% CI was 1.4-1.6 [36]. Thus, cigarette smokers were 1.5 times more likely to have PC than people who had never smoked. This study also showed that the risk of PC increased with an increased number of cigars consumed in a day while the OR was 1.82 for those who consumed more than 10 g of tobacco [36]. The OR was 1.1 while the 95% CI was 0.69-1.6 for pipe smokers. The OR for those who use smokeless tobacco compared with non-tobacco users was 0.98 while the 95% CI was 0.75-1.3. Therefore, this study did not show any statistical association between pipe smoking and smokeless tobacco with PC.

The amount of tobacco use can increase the chances of PC. Villeneuve et al. performed a case-control study to investigate the association between tobacco smoke and PC in Canada with 583 PC cases and 4,813 population-based controls between 1994 and 1997, including both children and adults. They reported an OR of 1.21 with a 95% CI of 0.60-2.44 in tobacco users compared to non-tobacco users [37]. Hence, tobacco use results in a dose-related increased risk for PC onset [38]. Former smoking was not statistically associated with the risk of PC. Talamini et al. conducted “ a case-control study in Italy between 1991 and 2008, including 326 cases and 652controls, with a median age of 63” [38]. A study was published in 2010 that reported that PC was associated with current smoking with an OR of 1.68 and a 95% CI of 1.13-2.48. Moreover, the risk increased in people who consumed more cigarettes daily. The OR was 2.04 with a 95% CI of 1.14-3.66 for those who smoked more than 20 cigarettes in one day [38]. There was no association found for former smokers, as the OR was 0.98 while the 95% CI was 0.66-1.45 [39]. Tobacco was observed to reduce the age of onset in sporadic PC [40].

Prolonged duration and increased amount of smokeless tobacco were also associated with the risk of PC [41]. Alguacil et al. conducted a case-control study of PC based on population from 1986 to 1989 in Georgia, Michigan, New Jersey, Detroit, and Atlanta [41]. This study included 526 patients with PC and 2153 controls between 30-79 years old. Participants who smoked cigars consistently showed a 70% elevated risk with a 95% CI of 0.9-3.3, and those who never used other forms of tobacco had a 90% elevated risk with a 95% CI of 0.8-4.3 [41]. The risk was increased among those who consumed >1 cigar in a day with an OR of 1.8 and 95% CI of 0.8-4.2, and those who consumed cigars for more than 20 years showed an OR of 1.9 with a 95% CI of 0.9-3.9 [41]. Trends depending on the quantity and total time smoked were apparent but not statistically significant, as the P-value was 0.17 and 0.16, respectively [34]. Smokeless tobacco users consistently had a 40% elevated risk of PC with 95% CI: 0.5-3.6 compared with non-users of tobacco [41]. The more the smokeless tobacco consumption, the more the chances of PC, as the P-value was 0.04. Furthermore, participants who consumed more than 2.5 oz of smokeless tobacco a week showed an OR of 3.5 with a 95% CI: 1.1-11 [41]. Chronic smokeless tobacco users (>20 years) had an OR of 1.5 with 95% CI: 0.6-4.0, which is an increased risk for PC but is not statistically significant [41]. In contrast, there was no increased risk in pipe smokers, as OR was 0.6 with 95% CI: 0.1-2.8 [41]. These studies showed that chronic smokeless tobacco users and, to a lesser extent, cigar smokers might raise the risk of PC among cigarette non-smokers [41]. Black tobacco was more significantly associated with PC than blonde tobacco. Molina-Montes et al. conducted a case-control study published in 2020 that included 2,009 cases and 1,532 controls (OR: 1.72; 95% CI = 1.39-2.12) [42]. They found that those who inhaled to their throat, chest and those who used non-filtered cigarettes had OR of 1.48 with 95% CI: 1.11-1.99,1.33 with 95% CI: 1.12-1.58, 1.69 with 95% CI: 1.10-2.61, respectively; all had an increased risk of PC [42]. Current black tobacco smokers had an increased risk for PC (OR was 2.09 with 95% CI: 1.31-3.41 compared to blond tobacco smokers (OR was 1.43 with 95% CI: 1.01-2.04) [42].

Compared to parental smoking, kid exposure to tobacco smoke was also linked with an elevated incidence of PC as OR was 1.24 with 95% CI: 1.03-1.49 [42]. Smokeless tobacco items are used worldwide, with rising prevalence in the United States and Northern Europe [43-45].

Different relevant studies that depict the correlation between smoking (tobacco) with PC are summarized in Table 2.

**Table 2:** Characteristics of included studies and patients

References	Design	Cases	Controls	Population	Results
Molina-Montes et al. (2020) [42]	Case-control study	2,009	1,532	Newly diagnosed patients with PC >18 years old and controls matched	OR: 1.72; 95% confidence interval (95% CI), 1.39-2.12
Bertuccio et al. (2011) [36]	Meta-analysis	6,056	11,338	All adults > 20 years of age	OR for cigar smokers was 1.6 as Compared with non-tobacco users, but while for cigarette smokers, the OR was 1.5
Talamini et al. (2010) [40]	Case-control study	326	652	The median age of 63 in Italy	OR for current smoking was 1.68 with 95% CI: 1.13-2.48 when the number of cigarettes per day increased OR was 2.04 with 95% CI: 1.14-3.66
Iodice et al. (2008) [35]	Meta-analysis			included 82 published studies	OR for current smokers was 1.74 with 95% CI 1.61-1.87, and OR for former smokers was 1.2 with 95% CI 1.11-1.29
Villeneuve et al. (2004) [37]	Case-control study	583	4,813	Includes both Children and Adults In Canada	OR was 1.21 with 95% CI: 0.60-2.44
Alguacil et al. (2004) [41]	Case-control study	526	2,153	Between the age of 30-79 years “among Atlanta, Georgia, Detroit, Michigan, and New Jersey residents."	Smoking cigars regularly shows a 70% elevated risk with 95% CI: 0.9-3.3, and those who never consumed any form of tobacco had a 90% elevated risk with 95% CI: 0.8-4.3

Impact of smoking cessation on PC occurrence and prognosis

Smoking cessation lowers the chances of PC. However, it requires more than a decade to lower the risk to that of never smokers [35]. Iodice et al. conducted studies about smoking cessation and risk reduction. People that had stopped smoking within the previous 10 years had a relative risk (RR) of 1.48 with 95% CI ¼ 1.25-1.76 while those who had quit smoking 10 and 20 years prior had a decreased RR of 1.15 with 95% CI ¼ 0.95-1.40 and 0.96 with 95% CI ¼ 0.85-1.09, respectively [35]. The risk in ex-smokers did not reach that of never smokers until more than 15 years after quitting smoking, according to the PanScan cohort consortium's pooled study [35]. Mulder reported a 45% and 30% decline in smoking prevalence among males and females in the European Union (EU) [46]. The predicted number of new patients with PC in the EU up to 2015 might be decreased by 15% if all current smokers immediately stopped smoking (around 150,000 patients) [46]. Realistically, this number would be more likely to be around 29,500 male and 9500 female patients [46]. A recent study that compared never smokers to past smokers for at least 10 years showed RR of 1.15 with 95 % CI: 0.95-1.40, a non-significantly increased risk of PC for past smokers for at least 10 years, and a non-significantly decreased risk of PC for those who had quit at least 20 years ago [35]. Participants who had not smoked for 15 years were at the same risk as never smokers in the above prospective study, regardless of the reference group [35]. Lin conducted a cohort study that showed a significantly decreasing trend in risk as to the number of years after smoking cessation increased (p-value = 0.04) among male ex-smokers. The RR was 0.85 (0.36-2.0) and 0.85 (0.36-2.0) for those who had stopped smoking for 10-19 and ≥20 years, respectively [47]. Because CS is one of the modifiable risk factors for PC, we hypothesize that successful primary prevention of PC should begin with quitting smoking [48]. PC mortality and morbidity are less common in western Europe with improved smoking cessation programs than in eastern Europe [49].

## Conclusions

This review has shown that tobacco is a strong risk factor for PC, leading to progressive fibrosis of pancreatic glandular tissue and stroma. Research into PC has become increasingly important in recent decades because of its increasing incidence and poor prognosis. In summary, this review has highlighted the detailed molecular pathogenesis of tobacco on pancreatic glandular tissue in terms of the epidemiology, clinical features, and management of PC. This review has evaluated important published data on the impact of nicotine on various disorders in people and the possible effects of nicotine on pancreatic pathogenesis in animal studies. PC would be significantly reduced if aggressive public health efforts to decrease smoking were implemented. We hope that this paper can help overcome these obstacles by outlining the pathophysiology, clinical symptoms, and management choices for PC. We briefly explored the challenges that physicians face when understanding the various associations between tobacco and PC. Furthermore, the incidence of PC can be reduced with smoking cessation and early screening, and the prognosis improved with proper management according to the clinical features, stage, and grade of PC. Further epidemiological and pathogenic research on PC should be conducted to help develop cancer control measures.
